# A rare case of left inferior vena cava presenting with May-Thurner syndrome

**DOI:** 10.1186/s42155-022-00305-2

**Published:** 2022-07-01

**Authors:** Jamal Moosavi, Parham Sadeghipour, Bahram Mohebbi, Kiara Rezaei-Kalantari, Ehsan Khalilipur

**Affiliations:** Cardiovascular Intervention Research Center, Rajaie Cardiovascular Medical and Research Center, Iran University of Medical Scences, Tehran, Iran

## Abstract

**Background:**

May–Thurner anomaly is characterized as external venous compression by the arterial framework against hard bony structures. This chronic anatomical lesion infrequently leads to deep vein thrombosis in the lower extremity, and it may lead to leg swelling as a long-term post-thrombotic complication. Left iliac vein compression may not be as uncommon as was previously thought, and it typically occurs in women more than men. Congenital anomalies of venous tree are not rare; they exist in 8.7% of the general population.

**Case-presentation:**

We herein present the first case of right-sided May Thurner Syndrome in a patient with IVC anomalies. In our patient, both common iliac veins formed the left-sided IVC, which extended to the hemiazygos vein and the superior vena cava. Additionally, there was a right-sided suprarenal IVC, which extended to the right atrium.

**Conclusion:**

Understanding the proper anatomy in May-Thurner syndrome helps in better decision making for management of disease pathophysiology.

## Introduction

May–Thurner anomaly is characterized as external venous compression by the arterial framework against hard constructions (Dogan and Boke 2005; Kibbe et al. 2004). This starts a confined central stenosis of the common iliac vein. Since compression in most scenarios happens against lower lumbar vertebrae, MTS should be suspected in patients with scoliosis or widened periumbilical veins (Steinberg and Jacocks 1993a; Moudgill et al. 2009). This chronic anatomical lesion infrequently leads to deep vein thrombosis (DVT) in the lower extremity, and it may result in leg swelling as a long-term post-thrombotic complication (Moudgill et al. 2009; Oteros Fernandez et al. 2008; Murphy et al. 2009; Patel et al. 2000). Frequently, iliac vein compression is left-sided; nonetheless, in some infrequent presentations, it involves the right-sided venous structures (Spentzouris et al. 2014; Ang et al. 2013). Congenital anomalies of the venous tree are not rare in that they exist in 8.7% of the general population (Truty and Bower 2007). It is particularly imperative to detect an IVC anomaly before vascular procedures or other interventional strategies because sudden entanglements can emerge (Mano et al. 2004).

Herein, we present the first case of MTS in a patient with IVC anomalies. In our patient, both common iliac veins formed the left-sided IVC, which extended to the hemiazygos vein and the superior vena cava (SVC). There was also a small right suprarenal IVC in the normal position with 1 retroaortic right renal vein. These anomalies led to an atypical MTS and compression in the right-sided venous system which crossed to the left and into the IVC.

## Case presentation

The patient was a 43-year-old woman with recurrent but non-documented right leg DVT during past 13 years. She used intermittent oral anticoagulation some years after first episode of her DVT and she complained of worsening right leg swelling, pain, formation of new varicose veins, and fatigue.

She had no specific medical or surgical illness and she have had 2 successful and uneventful pregnancies with normal vaginal delivery in spite of poor compliance to medical surveillance and lack of anticoagulant regimen during pregnancy period.

The patient was referred to our endovascular clinic for further assessment. In her physical examination, the right lower extremity was edematous with some hyperpigmentation and.

telangiectasia on her right leg. Additionally, prominent varicose veins were noticed on the right side. There were no active or healed ulcers on either lower extremity. Other cardiovascular examinations were unremarkable especially in terms of right-sided heart failure. The Villalta score for the further assessment of post-thrombotic syndrome based on its latest variables was 12, indicating a moderate post-thrombotic syndrome score (Kahn 2009; Kahn et al. 2009).

**Fig. 1:**
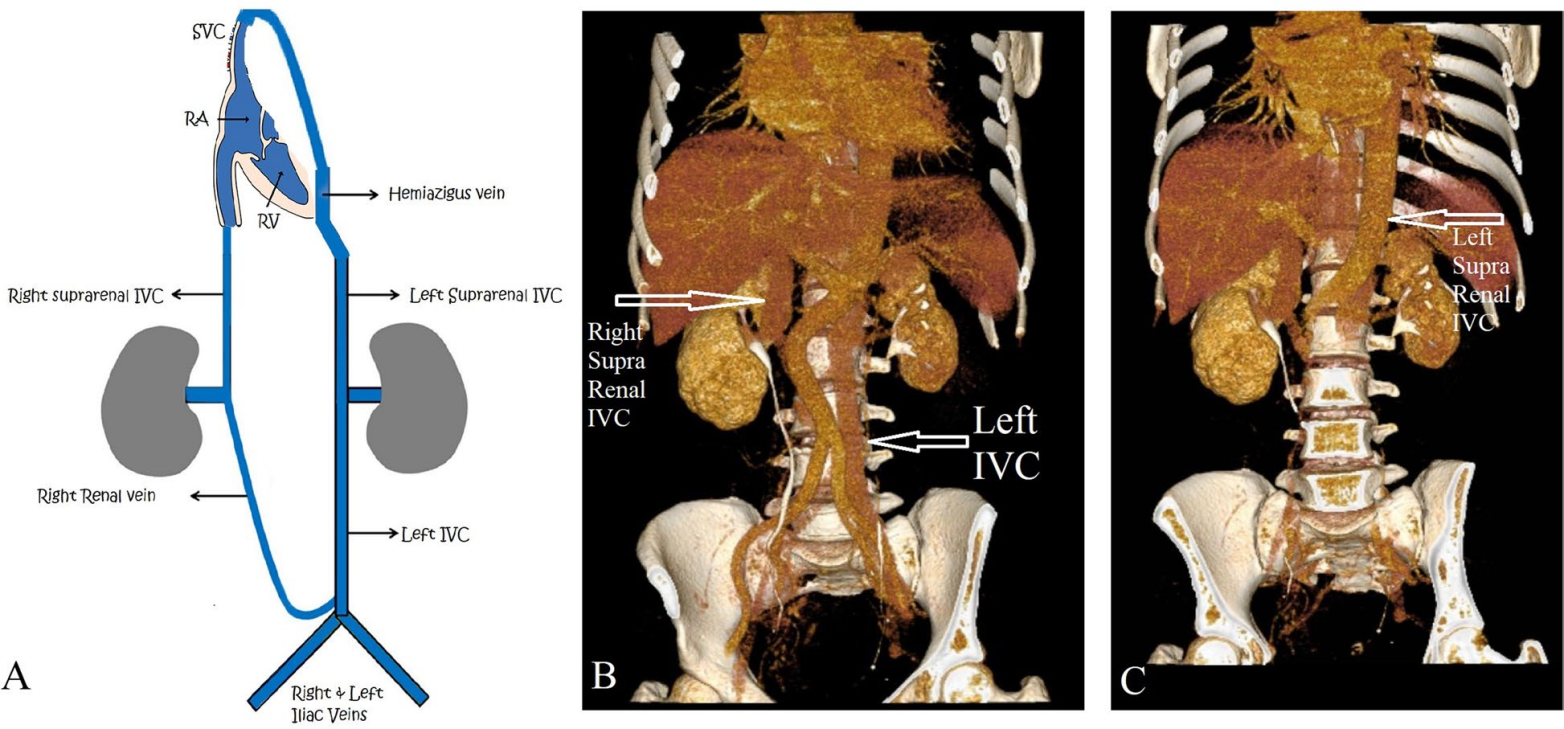
The image **A** illustrates the schematic anatomy of the patient’s IVC and **B**, **C** are reconstructed images of patient’s CT venography, IVC, Inferior vena cava; RA, Right atrium; RV, Right ventricle; SVC, Superior vena cava

The patient underwent a Doppler ultrasonography of both lower extremities, which revealed chronic post-thrombotic changes in the deep venous system with evidence of blunted pulse-Doppler waves of the right-sided veins. For detailed imaging of these findings, we had a computed tomography (CT) venography of both lower extremities depicted (Fig. 1).

**Fig. 2:**
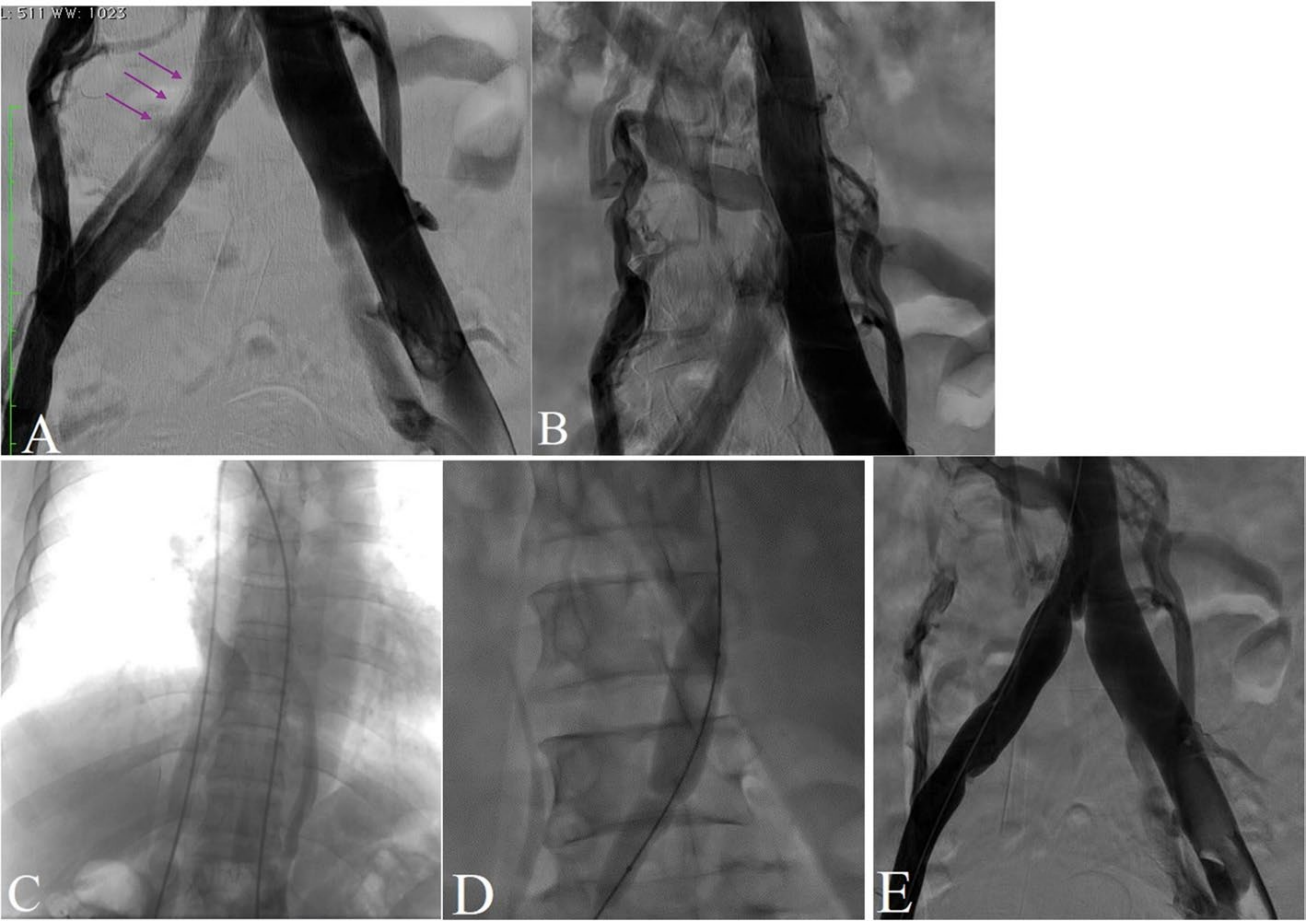
The images depict the patient’s venography (upper images) and venoplasty (lower images). In figure “**A**” the arrow shows compression on right iliac vein and in “**B**” the IVC anomaly is imaged and in “**C**” the wire path shows the connection of left sided IVC with hemiazygus vein and in “**D**” we perfomed a prolonged balloon inflation and “E” demonstrates the final acceptable angioplasty result

Unpredictably, the reconstructed image showed chronic post-thrombotic changes in the right lower extremity including right femoro-popliteal veins with the formation of a left-sided IVC after the union of both iliac veins, which extended to the hemiazygos vein. As the patient’s symptoms persisted enduringly, we decided to perform venography to determine the precise pathology of these changes. Preprocedural cardiac evaluations showed no abnormal echocardiographic or electrocardiographic findings. As is shown in Fig. 2, the lower extremity venous system after the iliac veins formed the left-sided IVC. Chiming in with the CT venography findings, the left-sided IVC extended to the hemiazygos vein and terminated at the SVC. On the other side, there was a suprarenal right-sided IVC, which joined the hepatic venous system and finally drained into the right atrium. There was also some evidence of compression on the right iliac vein and the proximal part of the left-sided IVC, suggesting the atypical MTS as the main cause of the patient’s chronic symptoms. We decided to perform balloon venoplasty for symptom relief and we deferred the stenting for next session if the patient symptoms does not relieve with venoplasty and compression therapy. The final venography demonstrated the acceptable outflow of both IVCs. The balloon venoplasty of the compressed venous system improved our patient’s venous hypertension as was reflected by improvements in both her subjective symptoms and her Villalta score. The patient was discharged the following day with oral anticoagulants, and she was asked to return for a follow-up the next month for the final approach like stenting if her symptoms persist.

## Discussion

In the most common variant of left iliac vein compression as a venous anomaly, it is mostly asymptomatic and because of this innocent presentation, it is usually underdiagnosed. In its symptomatic type, hypertension in the left lower extremity venous system occurs because of compression by the overriding iliac artery with or without left iliofemoral DVT (Suwanabol et al. 2010). Manifestations include left leg swelling, pain, and skin manifestations of venous stasis or thrombosis due to the mechanical venous obstruction of the left iliac vein by the right iliac artery and the underlying vertebral body. Such manifestations occur secondary to intimal hyperplasia from the repeated trauma, leading to venous narrowing due to intraluminal webs and fibrosis (Kibbe et al. 2004). MTS is mostly asymptomatic, which renders an accurate incidence estimation of this anomaly challenging. MTS may not be as uncommon as was previously thought, and it typically occurs in women more than men (Kim and Porter 1992).

MTS or iliac vein compression syndrome (IVCS) is not always simple compression, but sometimes it constitutes additional evidence of venous scarring and associated vessel changes, leading to symptoms of persistent venous hypertension (Steinberg and Jacocks 1993b). The clinical presentation consists of signs and symptoms of acute (52%) or chronic DVT, and the findings are often attributable to venous hypertension. The most prevalent findings are edema (90%), venous dilations (33.5%), persistent discomfort (89.7%), and recurring ulcers (5.1%). PTS (symptoms and signs of chronic venous insufficiency) is a result of the extension of the MTS sequeale and the postponement of its invasive treatment, since medical therapy alone is mostly insufficient for long-term maintenance (Hulsberg et al. 2016).

Various variants of MTS have been reported in the literature as atypical forms of this syndrome: compression of the left common iliac vein by the left internal iliac artery, compression of the right common iliac vein by the right internal and right common iliac arteries, and compression of the IVC by the right common iliac artery (Molloy et al. 2002; Fretz and Binkert 2010).

Kim et al. (Kim et al. 2018) were first to present a unique congenital anomaly of the IVC in which a left interrupted IVC extended to the hemiazygos vein and then the SVC before it drained into the right atrium. They described the anomaly, in which case both common iliac veins formed the left-sided IVC. Another component of the anomaly was a small right suprarenal IVC in the normal position. One retroaortic right renal vein formed the communicating point between the larger left IVC and the smaller right suprarenal IVC. Notably, the reported rare double IVC anomaly by Kim and colleagues had no more pathology and disease; however, unfortunately, our patient developed an unusual and atypical form of IVCS. Her right iliac vein was compressed between the left iliac artery and the spine, and she experienced mild-to-moderate post-thrombotic symptoms after some episodes of DVT.

The stenting of MTS is deemed safe, efficacious, and durable in both post-thrombotic patients and those treated for edema alone (Oteros Fernandez et al. 2008; Hager et al. 2013). Because stenting for MTS has been shown to have low morbidity and good patency with relief of symptoms, any patient with symptoms and identifiable iliac vein compression should be considered for stenting. The decision for intervention should be driven primarily by symptoms of venous outflow obstruction and guidance by history and physical examination. History of DVT may also increase the clinical indication for stenting (Salahuddin and Armstrong 2020; Kalantar et al. 2019). The balloon venoplasty of the compressed venous system improved our patient’s venous hypertension as was reflected by improvements in both her subjective symptoms and her Villalta score. We decided to follow our patient next weeks to assess angioplasty alone result. The mild nature of post-thrombotic symptoms in our patient based on the Villalta score prompted us to manage her without stenting and reserve the more definitive endovascular approach if the symptoms persisted or worsened (Kahn et al. 2009).

## Conclusion

Understanding proper anatomy in May-Thurner syndrome as a frequent venous disease is fundamental. In our case, a very rare variation accompanied the patient’s symptoms and it was obviated by balloon angioplasty. Also these patients need a careful surveillance to diagnose future symptoms earlier.

## Data Availability

All data are available if need for publication.

## Abbreviations

MTS: May-Thurner syndrome
IVC: Inferior vena cava
DVT: Deep vein thrombosis
SVC: Superior vena cava
CT: Computed tomography
RA: Right atrium
RV: Right ventricle
SVC: Superior vena cava
IVCS: Iliac vein compression syndrome
